# Dual action of epigallocatechin-3-gallate in virus-induced cell Injury

**DOI:** 10.1186/s43141-023-00624-4

**Published:** 2023-11-28

**Authors:** Ahmed Mostafa, Gomaa Mostafa-Hedeab, Hany Abdelfattah Elhady, Esraa Ahmed Mohamed, Abozer Y. Eledrdery, Sager Holyl Alruwaili, Ahmed Mohamed Al-Abd, Abdou Kamal Allayeh

**Affiliations:** 1 Disease Intervention and Prevention Program, Texas Biomedical Research Institute, San Antonio, TX 78227 USA; 2 Center of Scientific Excellence for Influenza Viruses, National Research Centre, Giza, 12622 Egypt; 3 Pharmacology Department and Health Research Unit, Medical College, Jouf University, 11564 Skaka, Saudi Arabia; 4 Surgery Department, Medical College, Jouf University, 11564 Sakaka, Saudi Arabia; 5 Virology Department, Nawah Scientific Co, Almokattam Mall, Street 9, Egypt, 11562 El Mokattam Egypt; 6 Department of Clinical Laboratory Sciences, College of Applied Medical Sciences, Jouf Uni-Versity, 11564 Sakaka, Saudi Arabia; 7 Department of Surgery, Orthopedic Division, College of Medicine, Jouf University, 11564 Sakaka, Saudi Arabia; 8 Pharmacology Department, Medical and Clinical Research Institute, National Research Centre, Giza, 12622 Egypt; 9 Water Pollution Department, Virology Laboratory, National Research Centre, Dokki, 12622 Giza Egypt

**Keywords:** Green tea extract, EGCG, COVID-19, Antiviral activity, Wound healing, Herpes simplex virus, Cellular injury

## Abstract

**Background:**

Viral infections cause damage and long-term injury to infected human tissues, demanding therapy with antiviral and wound healing medications. Consequently, safe phytochemical molecules that may control viral infections with an ability to provide wound healing to viral-induced tissue injuries, either topically or systemically, are advantageous. Herein, we hypothesized that epigallocatechin-3-gallate (EGCG), the most abundant polyphenol in green tea, might be effective as a wound healing, antiviral, and antifibrotic therapy.

**Results:**

The antiviral activities of EGCG against severe acute respiratory syndrome coronavirus 2 (SARS-CoV-2) and Herpes simplex virus type 2 (HSV-2) as well as its wound healing activities against different monolayer tissue (continuous and primary) systems were investigated. Consider its possible wound-healing advantages as well. To determine the safe concentrations of EGCG in green monkey kidney (Vero) and Vero-E6 cell lines, MTT assay was performed and showed high CC_50_ values of 405.1 and 322.9 μM, respectively. The antiviral activities of EGCG against SARS-CoV-2 and HSV-2, measured as half-maximal concentration 50 (IC_50_) concentrations, were 36.28 and 59.88 μM, respectively. These results confirm that the EGCG has remarkable viral inhibitory activities and could successfully suppress the replication of SARS-CoV-2 and HSV-2 in vitro with acceptable selectivity indices (SI) of 11.16 and 5.39, respectively. In parallel, the EGCG exhibits significant and dose/time-dependent anti-migration effects in human breast cancer cells (MCF-7), its resistant variation (MCF-7^adr^), and human skin fibroblast (HSF) indicating their potential to heal injuries in different internal and topical mammalian systems.

**Conclusions:**

The EGCG has proven to be an efficient antiviral against SARS-CoV-2 and HSV-2, as well as a wound-healing phytochemical. We assume that EGCG may be a promising option for slowing the course of acute cellular damage induced by systemic (Coronavirus Disease 2019 (COVID-19)) or topical (HSV-2) viral infections.

## Background

Antiviral therapy is the first control option to consider once a virus infection is established. All the steps in the virus life cycle ranging from entry to release can be explored as molecular targets for specific antiviral therapy as well as virus-dependent cell targets for indirect antiviral therapy. Moreover, antiviral therapy should certainly be used if patients are at high risk for tissue injury via virus-induced cellular responses or if patients are immunocompromised.

SARS-CoV-2 is one of many viruses that infect the human respiratory system, causing symptoms ranging from modest upper airway involvement to life-threatening acute respiratory distress syndrome (ARDS). Lung consequences, as illustrated by the current COVID-19 pandemic, include pneumonia and acute respiratory distress syndrome (ARDS) in critical cases [1, 24, 52]. Due to the combined effect of direct viral and indirect patient-specific immune-mediated damage, the clinical picture of lung tissue destruction is difficult to anticipate. It is widely known that SARS-CoV-2 interacts with the angiotensin-converting enzyme 2 (ACE2), which is predominantly found in type II pneumocytes in the lungs. ACE2 expression changes as a result of viral binding appear to be associated with increased vascular permeability, increased lung edema, increased lung injury, and overproduction of proinflammatory factors [16, 29]. In clinical terms, the lungs become irritated and filled with fluid, resulting in breathing difficulties. Breathing issues in certain individuals can become serious enough to require hospitalization with an oxygen ventilator. Consequently, lung injury may take months to improve. Another potential COVID-19 consequence is sepsis, which occurs when an infection spreads through the circulatory system, causing tissue damage everywhere it goes. Sepsis, even if a patient survives, can cause long-term damage to the lungs and other organs [18–20].

On the other hand, Herpes simplex virus (HSV) is another prevalent viral disease globally. HSV type-1 (HSV-1) infection occurs in or around the mouth and is mostly spread by oral-to-oral contact, whereas HSV type-2 (HSV-2) infection occurs sexually and causes genital herpes [42]. Both oral and genital herpes are usually asymptomatic. Nevertheless, they can cause painful blisters or ulcers, ranging from moderate to severe [4]. Although herpesviruses do not primarily target the lungs,under certain circumstances, several of them can cause interstitial pneumonia, bronchopneumonia, and ARDS. Infected persons commonly report tingling, stinging, or burning around their lips prior to the onset of sores. These symptoms may return regularly, with the frequency changing depending on the individual [32]. Besides, HSV infects the lower respiratory tract (LRT) of immunocompetent and immunocompromised patients [11, 9]. For instance, in many burned or intubated patients with squamous metaplasia of the respiratory epithelium, the spreading of the virus to the lung is probably an extension or aspiration of oropharyngeal HSV, or via hematogenous spread [11].

In any case, the rising trend in the prevalence of viral diseases drives researchers to explore treatment solutions that not only inactivate the virus but also aid in the process of regenerating wounded cells in order to mitigate the cellular damaging consequences of viral infection.

Natural flavonoids, a diverse set of polyphenolic substances found in plants, should be explored as a potential treatment for viral infections. For instance, the polyphenol epigallocatechin-3-gallate (EGCG), a major active ingredient in green tea, has been shown to have anti-inflammatory [35], antioxidant [10], anti-fibrotic [37], antimicrobial [53] and antiviral activities [51]. In this study, we investigated the possible use of EGCG to control viral infections with RNA and DNA model viruses of known viral-induced cell injuries. The antiviral activity against pandemic SARS-CoV-2 and HSV-2 and the possible wound-healing activity following the induction of cell-monolayer injuries in various mammalian systems were studied.

## Results

### The cytotoxicity (CC50) of EGCG on Vero and Vero-E6 cell lines

**Fig. 1:**
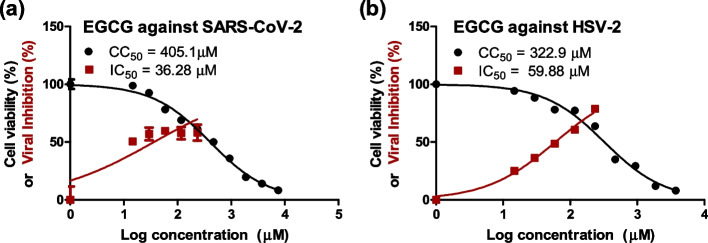
Cytotoxicity and antiviral activity of EGCG against SARS-CoV-2 (**a**) and HSV-2 (**b**). The Cytotoxicity and antiviral activity were determined in Vero E6 and Vero cells against NRC-03-nhCoV and HSV-2, respectively. Half maximal cytotoxic (CC_50_) and inhibitory (IC_50_) concentrations were calculated using nonlinear regression analysis of GraphPad Prism software (version 5.01) by plotting log inhibitor versus normalized response (variable slope)

The EGCG was serially two-fold diluted and added to the cell culture medium to examine its effect on the growth and viability of Vero and Vero-E6 cell lines. After 3 days of co-incubation, the cell viability of Vero and Vero–E6 cells was determined using MTT assay. The mean dose–response curve of three different experiments was used to calculate the 50% cytotoxic and growth inhibition doses. The half-maximal cytotoxic concentrations of EGCG was determined to be 322.9 and 405.1 μM in Vero and Vero-E6 cells, respectively (Fig. 1).

### *EGCG exerts antiviral activity against SARS-CoV-2 and HSV-2 *in vitro

The cytopathic inhibition experiment was used to investigate EGCG's antiviral activity against SARS-CoV-2 and HSV-2. As a control, untreated virus-infected cells were employed in the test. The 50% inhibitory concentration (IC_50_) values for SARS-CoV-2 and HSV-2 were determined to be 36.28 and 59.88 μM, respectively (Fig. 1).

The selective indices are calculated by dividing the CC_50_ by IC_50_ (SI = CC_50_/IC_50_) values and are found to be 11.16 and 5.39 for SARS-CoV-2 and HSV-2, respectively (Table 1). At non-cytotoxic EGCG concentrations, SARS-CoV-2 infectivity was reduced by more than 59%, while HSV-2 infectivity was decreased by 41%.

**Table 1:** Inhibitory Concentration (IC_50_) and selective index (SI) for EGCG against both viral infections

Sample	Cell	CC_50_ (μM)	Virus	IC_50_ (μM)	SI
EGCG	Vero-E6	405.1	SARS-CoV-2	36.28	11.16
	Vero	322.9	HSV-2	59.88	5.39

### Stage of antiviral activity

**Fig. 2:**
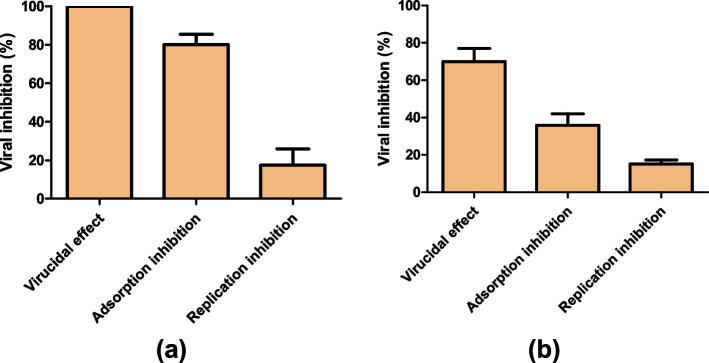
Stage of antiviral activity of EGCG. The EGCG (50 μM) has been tested against SARS-CoV-2 (**a**) and HSV-2 (**b**) at different stages of the viral replication cycle including cell-free status “virucidal effect”, adsorption inhibition, and interference with viral replication

To confirm whether the anti-SARS-CoV-2 and anti-HSV-2 activity of EGCG can be attributed to the inhibition of the virus in a cell-free status or virus adhesion to the host cell receptors or replication inside the host cell, plaque reduction assays were performed as previously described [14]. Consequently, the EGCG affected both viruses mainly by targeting them in a cell-free status/virucidal effect (upto 100% inhibition of SARS-CoV-2 virus and 69.97 ± 7.1% of HSV-2), followed by interference with viral adsorption (average viral inhibition equals 80.1 ± 5.3% for SARS-CoV-2 and 35.1 ± 6.21 for HSV-2). The interference of EGCG with the replication efficiency of both viruses was low (average viral inhibition equals 17.44 ± 8.5% for SARS-CoV-2 and 15.03 ± 2.2% for HSV-2), when compared to the other two mechanisms or replication-cycle stages (Fig. 2 a & b).

### *EGCG exerts an anti-migration effect against MCF-7 and its resistant variant (MCF-7*^*adr*^*) cells*

**Fig. 3:**
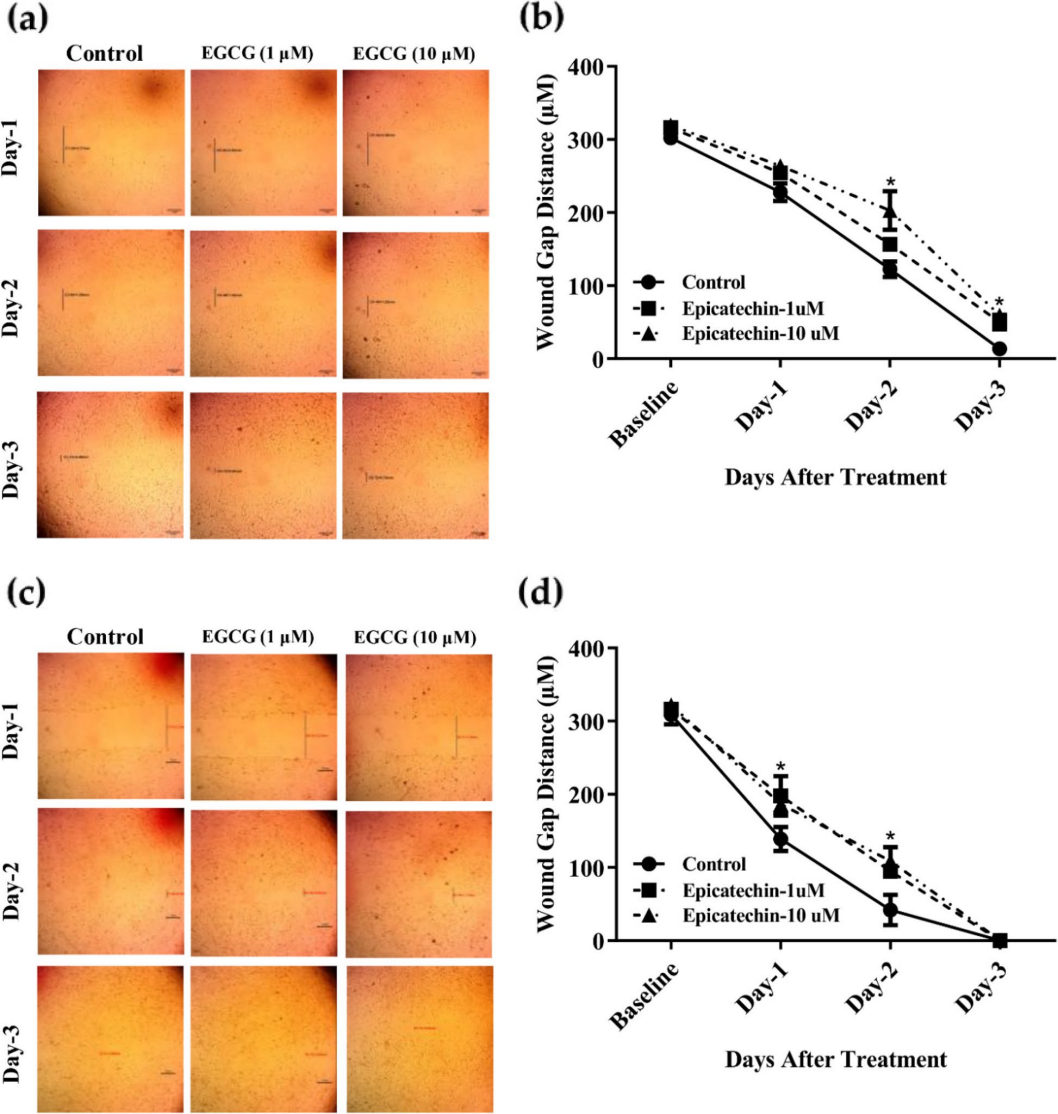
The effect of EGCG on MCF-7 and MCF-7^adr^ cell migration. MCF-7 and MCF-7^adr^ cell monolayer sheets were scratched and treated with EGCG (1 and 10 μM). Images for the wound gap distance were taken for treated and control cells after 24 h, 48 h, and 72 h (**a** & **c**). Wound gap distances were optically measured and displayed over time (**b** & **d**). Data are shown as mean ± SD; *n* = 3. *P < 0.05, versus the control group

The anti-migration effects of EGCG against MCF-7 and its resistant variant MCF-7^adr^ were assessed in-vitro using a wound healing assay to reflect the potential of EGCG in suppressing tumor invasion. MCF-7 and MCF-7^adr^ cells were treated with 1 μM and 10 μM of EGCG for 24 h (Day-1), 48 h (Day-2), and 72 h (Day-3), and wound closure was compared to the control untreated group. In MCF-7 cells, after 24 h treatment with EGCG, no significant change in wound gap was observed between treated and control cells. Further exposure of cells to EGCG significantly delayed wound closure compared to control untreated cells. After 48 h, EGCG (10 μM) showed a significant migration inhibition in MCF-7 cells (wound gap of 263.7 ± 9.3 μm) compared to control untreated cells with wound gap of 227.9 ± 17.0 μm. Prolonged exposure of cells to both 1 μM and 10 μM of EGCG for 72 h significantly delayed the cell migration with a wound gap of 156.3 ± 12.6 μm-wide and 202.9 ± 37.6 μm-wide, respectively, while the gap in control untreated cells was 122.3 ± 15.2 μm (Fig. 3 a & b).

In MCF-7^adr^ cells, EGCG significantly delayed wound closure compared to untreated control cells as early as after 24 h exposure. Wound gap distances were found to be 197.6 ± 38.5 μm-wide and 186.3 ± 17.1 μm-wide after treatment with 1 μM and 10 μM of EGCG, respectively, compared to wound gap of 138.6 ± 23.4 μm for untreated control cells. After 48 h, EGCG (1 μM 10 μM) showed further significant migration inhibition with a wound gap of 94.6 ± 13.9 μm-wide and 108.6 ± 26.9 μm-wide, respectively, while the gap in control untreated cells was 41.6 ± 29.4 μm (Fig. 3 c & d). Therefore, it can be concluded that EGCG exhibited a significant dose/time-dependent anti-migration effect against MCF-7 cells and its resistant variant (MCF-7^adr^).

### EGCG facilitates wound healing effect using normal human skin fibroblast cells

**Fig. 4:**
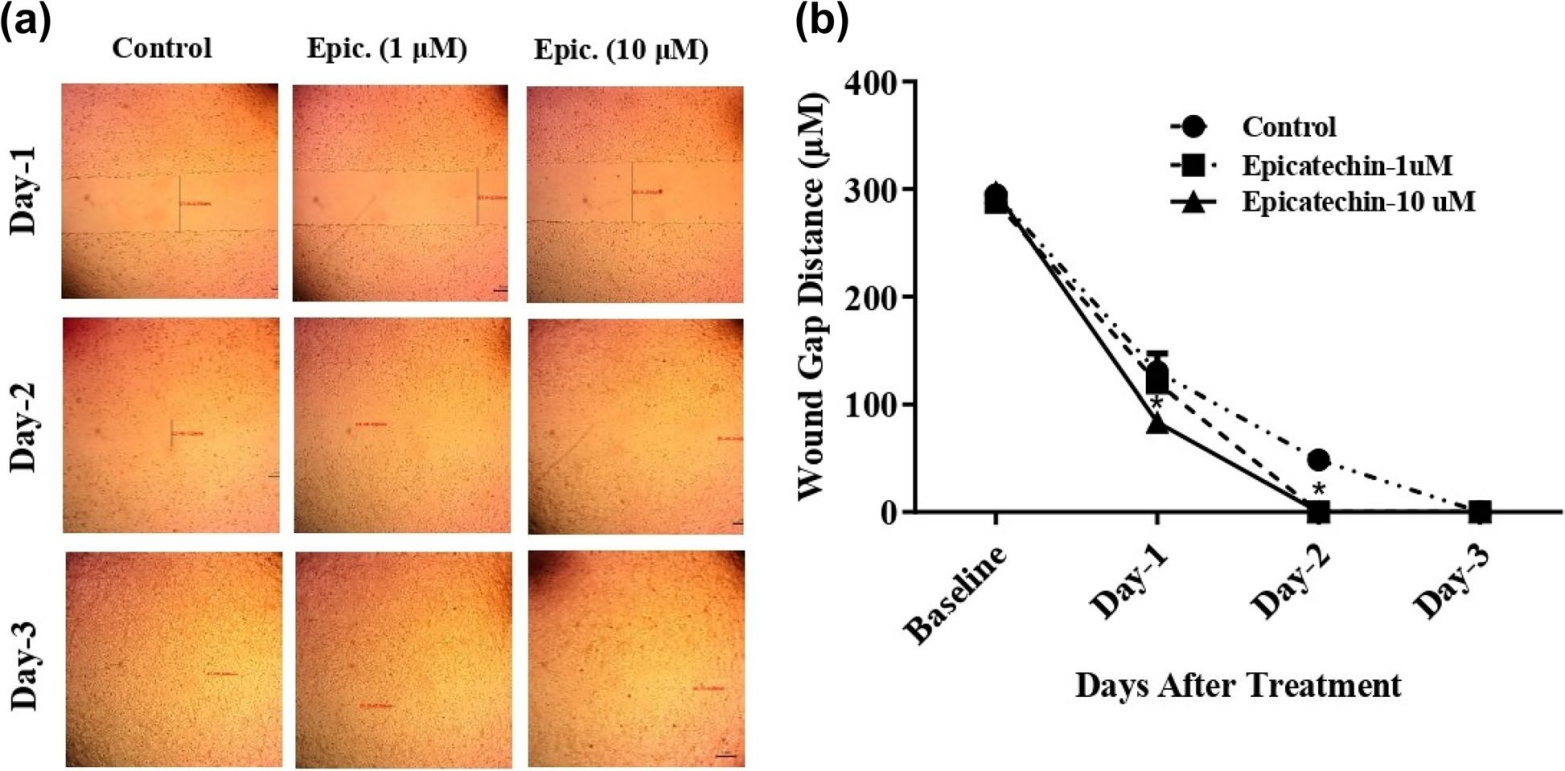
The effect of EGCG on HSF cell migration. HSF cell monolayer sheets were scratched and treated with EGCG (1 and 10 μM). Images for the wound gap distance were taken for treated and control cells after 24 h, 48 h, and 72 h (**a**). Wound gap distances were optically measured and displayed over time (**b**). Data are shown as mean ± SD; *n* = 3. *P < 0.05, versus the control group

Another common interpretation for the scratch assay is the wound healing capacity when the drug under investigation is tested against normal skin fibroblast cells. Herein, we investigated the wound healing capacity of EGCG (1 and 10 μM) against HSF cells (human skin fibroblast). EGCG (10 μM) enhanced the wound closure as early as after 24 h with a wound gap of 119.7 ± 3.3 μm compared to 130.0 ± 24 μm for untreated control fibroblast. After 48 h, both concentrations of EGCG (1 μM and 10 μM) showed a total wound closure compared to a remaining wound gap of 48.3 ± 9.8 μm in the untreated control fibroblast cells (Fig. 4 a & b). Therefore, and in contrast to cancer cells, EGCG facilitates wound healing in normal fibroblast cells in a dose and time-dependent manner.

## Discussion

Acute systemic and topical viral infections including COVID-19 and herpes are usually associated with cell injuries and wounds that persist for longer times after termination of viral replication and shedding [34]. In addition, the COVID-19 pandemic is meaningfully associated with many social factors including depression that affect immune fitness [21], delaying the wound healing process [3, 40].

Since the emergence of COVID-19, the interest is growing among researchers to develop effective antivirals to alleviate disease progression, reduce viral replication and infection spreading. Nevertheless, the pathological picture of COVID-19 is usually associated with diffuse alveolar damage (DAD) that demand precise and immediate healing [39]. The abnormal wound healing of this damage may result in additional severe scarring and fibrosis than other forms of the life-threatening ARDS [30]. The stepwise recovery of this damage demands a dynamic innate and acquired immune responses and epithelial cells regeneration and induction via wound-healing phytochemicals rather than administrating epithelial growth factors that are more likely to be detrimental and could increase the viral load via the upregulation of the ACE2 expression on the host cells [30].

On the same hand, Herpes simplex virus is capable of causing topical partial-thickness wounds [38]. Similarly to COVID-19, herpes simplex virus infection delays healing of oral excisional and extraction wounds in experimental animals [13]. To these points, we sought to identify a safe phytochemical molecule that can serve as antiviral medication with the ability to aid in healing the infection-mediated epithelial/endothelial injury.

Epigallocatechin-3-gallate (EGCG), a form of catechin, is a well-known natural antiviral drug with the potential to inhibit numerous viruses as well as reduce oxidative damage and promote lung regeneration capacity [27, 31, 33, 45]. The EGCG has an inhibitory impact on a wide variety of pathogens and functions as a broad-spectrum antiviral agent. It is most common in the following locations: (a) EGCG inhibits cytomegalovirus [22], Zika virus [5], and human immunodeficiency virus [12, 26] aggressively during early infection,(b EGCG inhibits viral replication, including influenza virus [23], enterovirus [15], and hepatitis virus [57],and (c EGCG inhibits pathogens indirectly by regulating immune inflammation and oxidative stress [55].

Herein, the wound healing and antiviral effects of EGCG on multiple cell lines, as well as two viral pathogens, COVID-19 and HSV-2, were accordingly investigated. In parallel, EGCG showed remarkable antiviral efficacy against SARS-CoV-2 (RNA virus) and HSV-2 (DNA virus) at safe concentrations. The data in this study confirmed that EGCG has high CC_50_ values reflecting its low cytotoxicity. EGCG has low inhibitory concentration 50 (IC_50_) against SARS-CoV-2 and HSV-2 (36.28 and 59.88 μM, respectively), indicating its remarkable antiviral activity against RNA and DNA model viruses. The investigation of the stage at which EGCG can impair viral replication cycle, it was found in this study that it affects the viral particle directly “virucidal effect” as well as interferes with the virus ability to adsorb to the host cell receptors. Consistently, this study confirms that EGCG exhibits broad antiviral activity against numerous viruses [25, 28, 36, 49, 54]. It has previously been shown to prevent the attachment of hepatitis viruses, influenza, reovirus, coronavirus and vesicular stomatitis virus by acting directly on the virions or interacting with surface proteins [8, 25, 58]. These prior studies back up our findings and indicate that EGCG inhibits the propagation of a variety of viruses, including RNA and DNA viruses. EGCG has also been proposed to inhibit 3CL-Protease of SARS-CoV-2 [17]. The IC50 value as an indicator of the viral inhibitory effect was found to be variable against different viruses including SARS-CoV-2 [28]. This shows that EGCG can hinder the infectivity of different viral pathogens, however the wide disparity in IC_50_ values could be due to a number of factors such as EGCG extraction technique, cellular and viral model variations, or antiviral methods used.

Furthermore, EGCG has been shown and confirmed to have a wide anti-lung fibrosis impact. To the best of our knowledge, there is no reported relationship between COVID-19 induced lung fibrosis and type of lung cancers [6, 50]. Pulmonary fibrosis, for instance, develops in COVID-19 patients because SARS-CoV-2 infection induces a massive increase in neutrophil infiltration into the lungs, leading to TGF production. An unregulated surge in active TGF-beta 1, aided by proinflammatory cytokines such as TNF, IL-6, and IL-1, induces fast and widespread edema and fibrosis and eventually clogs the airways resulting in lung functional failure [6]. Some studies investigated the protective impact of EGCG against lung fibrosis and found that it enhanced lysosomal hydrolases and ultrastructural changes in the lungs of a bleomycin-induced rat model of lung fibrosis [46–48]. Furthermore, downregulating TGF-β1 signaling inhibited fibroblast activation and collagen buildup, providing solid evidence that EGCG is an effective anti-fibrotic medication [6]. In line with previous findings, the documented antioxidant, anti-inflammatory, antimicrobial, angiogenesis and antifibrotic properties of the EGCG potentiate its activity at diverse stages of topical wound healing including hemostasis, inflammation, proliferation and tissue remodeling [56, 59].

Over and above, EGCG is known to possess potential chemopreventive as well as chemotherapeutic actions [7]. In the current study, EGCG showed significant antiproliferative as well as anti-invasive properties in both naïve (MCF-7) and resistant (MCF-7^Adr^) tumor cells. Breast adenocarcinoma cells were used herein as a proof of principle and to compare the potential effect of EGCG on sister naïve and resistant cell lines.

## Conclusions

To sum up, the potential inhibitory impacts of EGCG as an antiviral therapy option against SARS-CoV-2 and HSV-2 were confirmed in this study. In addition to its antiviral characteristics, EGCG has demonstrated potent and dose/time-dependent anti-invasion effects on malignant cell lines. On the top of these therapeutic effects, EGCG showed enhancement in wound healing properties on normal fibroblast cells which can cushion all previously mentioned effects’ collateral damages. This emphasizes that the EGCG can be further investigated in vivo in preclinical studies as a trial to be applied as a candidate potentially safe antiviral and wound-healing phytochemical in COVID-19 and HSV-2 infections.

## Methods

### Cell Culture, EGCG, and Viruses

Nawah Scientific Inc. (Mokattam, Cairo, Egypt) provided the African green monkey kidney (Vero), (Vero-E6), human breast cancer (MCF-7), doxorubicin-resistant breast cancer (MCF-7^Adr^), and human normal skin fibroblast (HSF) cell lines, which were cultured in DMEM (Dulbecco's Modified Eagle's Medium), Gibco, USA. The culture media was supplemented with 10% fetal bovine serum (FBS) and 100 units/mL penicillin/streptomycin (PS). The cells were incubated at 37 oC in a humid atmosphere with 5% CO_2_. Sigma-Aldrich (Seelze, Germany) provided the EGCG, which was diluted to stock solutions with PBS and kept at 80° C for all subsequent studies.

Nawah-Scientific Co. for Scientific Research Services, Egypt generously contributed HSV-2 for in vitro viral challenge. The hCoV-19/Egypt/NRC-3/2020 SARS-CoV-2 (NRC-03-nhCoV) was obtained from the virus collections of the Centre of Scientific Excellence for Influenza viruses at the National Research Centre, Egypt. The viral titers were calculated using the limit-dilution method and were expressed as a 50% cell culture infective dosage (TCID_50_) of 1 × 10^4^ (SARS-CoV-2), and 1 × 10^6^ (HSV-2), respectively. Virus stocks were kept at 80 °C until they were used.

### Cytotoxicity of EGCG on Vero and Vero-E6 Cell lines

Based on prior reports [41, 43], cells were seeded in a 96-well plate at a density of 2 × 10^5^ cells/well and then treated for 72 h at 37 °C in a humidified environment of 5% CO_2_ with two-fold concentrations of EGCG (7.5–0.0146 mM). Following the incubation period, the medium was replaced with 100 μl of MTT solution (5 mg/ml) and incubated at 37 °C for 4 h. After 30 min at 37 °C, the MTT solution was changed with 50 μl of acidified isopropanol. The absorbance at 570 nm was then measured to estimate the maximum concentration of EGCG that was not toxic to the cells using the next equation. (A-B/A) × 100 was used to calculate the 50% cytotoxic concentration (CC_50_), where A & B are the means of three OD_570_ measurements of untreated and treated cells, respectively.

### *Efficacy of EGCG against SARS-CoV-2 and, HSV-2 challenges *in vitro

To investigate the antiviral activity of EGCG against SARS-CoV-2 and HSV-2, the half maximal inhibitory concentration 50 (IC_50_) were estimated as previously described [14]. Briefly, Vero and Vero-2 confluent 96-well plates were infected for 60 min at 37 oC with 100 μl of stock SARS-CoV-2, and HSV-2 viruses. The EGCG was then added in 100 μl increments. Three wells were utilized for each dilution, and 100 μl of the maintenance medium was added to each well. Plates were finally incubated for three days until cytopathic effect (CPE) was observed. Subsequently, the cells were fixed with 100 μL/well 10% fixing solution and incubated for 2 h at room temperature, then the supernatants were discarded and 50 μl/well of 0.1% crystal violet stain were added for 10 min. The dried stained treated and control wells were then supplemented with 180 μl/well of absolute methanol and shaked for 30 min. The optical density (OD) was then measured at 570 nm using ELISA plate reader. A plot of cell viability (%) and viral inhibition (%) versus concentration for each EGCG was represented using GraphPad prism 5 software.

### Mode/Stage of antiviral action

To define the stage at which EGCG is affecting SARS-CoV-2 and HSV-2, three main stages of the viral replication cycle including (a) virucidal effect; (b) adsorption inhibition; and (c) replication interference, were investigated by plaque reduction assay of action as previously described [14]. The EGCG was applied in the three protocols at an effective concentration of 100 μM (< CC_50_ and > IC_50_ values). The percent of viral reduction was calculated using the following equation:

$$\mathbf{Plaque}\;\mathbf{reduction}\;(\boldsymbol{\%})=\frac{\mathbf{Count}\;\mathbf{of}\;\mathbf{untreated}\;\mathbf{virus}\;(\mathbf{control})-\mathbf{Count}\;\mathbf{of}\;\mathbf{treated}\;\mathbf{virus}}{\mathbf{Count}\;\mathbf{of}\;\mathbf{untreated}\;\mathbf{virus}\;(\mathbf{control})}\times100$$

### Wound healing assay

Wound healing assay (scratch assay) was used herein to assess the anti-migration effect of EGCG against MCF-7 (human breast adenocarcinoma cells), and its resistant variant MCF-7^adr^ and HSF (human skin fibroblast) cells. Briefly, 1 × 10^5^ cells were seeded in a 6-well plate and maintained until a minimum of 80% confluent monolayer cell sheet. Afterward, monolayer cell sheets were scratched with a sterile pipette tip to generate a 40 μm-wide wound, and scratched cell debris was washed out with PBS. Fresh media or media containing EGCG were added to cells and incubated for a further 72 h. Wound closure was monitored every day by collecting digitized images using TCM-400 inverted microscope (LaboMed, Fremont, CA, USA) coupled with a Digital Still Camera (35 mm SLR camera) for scratch width calculation [2].

### Statistical analysis

All experiments were performed in triplicate and calculations were carried out using GraphPad PRISM and linear regression analysis (Version 8.0.1, GraphPad Software, San Diego, CA, USA). The selective index (SI) was derived using CC_50_/IC_50_ [44].

## Data Availability

All data generated or analyzed during this study are included in this article.
